# Effectiveness of Common Household Cleaning Agents in Reducing the Viability of Human Influenza A/H1N1

**DOI:** 10.1371/journal.pone.0008987

**Published:** 2010-02-01

**Authors:** Jane S. Greatorex, Rosanna F. Page, Martin D. Curran, Paul Digard, Joanne E. Enstone, Tim Wreghitt, Penny P. Powell, Darren W. Sexton, Roberto Vivancos, Jonathan S. Nguyen-Van-Tam

**Affiliations:** 1 Health Protection Agency, Addenbrookes Hospital, Cambridge, United Kingdom; 2 Department of Pathology, University of Cambridge, Cambridge, United Kingdom; 3 Public Health and Epidemiology, University of Nottingham, Nottingham, United Kingdom; 4 Biomedical Research Centre, University of East Anglia, Norwich, United Kingdom; 5 Health Protection Agency, Cheshire and Merseyside Health Protection Unit, Cheshire, United Kingdom; 6 Health Protection Agency, East Midlands, Nottingham, United Kingdom; Institute of Molecular and Cell Biology, Singapore

## Abstract

**Background:**

In the event of an influenza pandemic, the majority of people infected will be nursed at home. It is therefore important to determine simple methods for limiting the spread of the virus within the home. The purpose of this work was to test a representative range of common household cleaning agents for their effectiveness at killing or reducing the viability of influenza A virus.

**Methodology/Principal Findings:**

Plaque assays provided a robust and reproducible method for determining virus viability after disinfection, while a National Standard influenza virus RT-PCR assay (VSOP 25, www.hpa-standardmethods.org.uk) was adapted to detect viral genome, and a British Standard (BS:EN 14476:2005) was modified to determine virus killing.

**Conclusions/Significance:**

Active ingredients in a number of the cleaning agents, wipes, and tissues tested were able to rapidly render influenza virus nonviable, as determined by plaque assay. Commercially available wipes with a claimed antiviral or antibacterial effect killed or reduced virus infectivity, while nonmicrobiocidal wipes and those containing only low concentrations (<5%) of surfactants showed lower anti-influenza activity. Importantly, however, our findings indicate that it is possible to use common, low-technology agents such as 1% bleach, 10% malt vinegar, or 0.01% washing-up liquid to rapidly and completely inactivate influenza virus. Thus, in the context of the ongoing pandemic, and especially in low-resource settings, the public does not need to source specialized cleaning products, but can rapidly disinfect potentially contaminated surfaces with agents readily available in most homes.

## Introduction

Influenza A virus poses a major public health problem and is associated with frequent annual epidemics and occasional pandemics. During annual epidemics, the disease is associated with excess mortality and morbidity (including hospitalisations), especially in the elderly, children under 2 years, and those of all ages with underlying high-risk co-morbidities. In April, 2009 a novel influenza virus (A/H1N1v) was identified in North America, but had already spread extensively in Mexico during the preceding weeks. Rapid global spread ensued and a global pandemic was formally declared by the World Health organization on June 11^th^ 2009.

During an influenza pandemic, the majority of people who develop symptoms will stay at home, where informal lay care will most often be provided by relatives. It is therefore crucial to gather specific information about how the virus is shed around the home and how transmission may be reduced by the adoption of appropriate hygiene measures, including cleaning of surfaces likely to be contaminated by virus. This is especially important in households containing young children as the latter are well known for their poor respiratory etiquette and higher virus shedding. Whilst numerous commercial virucidal agents are currently available, they may become scarce during a pandemic and are not available in low-resource settings. The purpose of this work was to assess a representative selection of simple, household cleaning agents and commercially available wipes which might be readily utilised to reduce the amount of virus spread around the home. We find that dilute solutions of washing up detergent, bleach or vinegar provide suitable means of disinfecting surfaces of influenza A virus.

## Results

### Liquid Cleaning Agents

**Figure 1:**
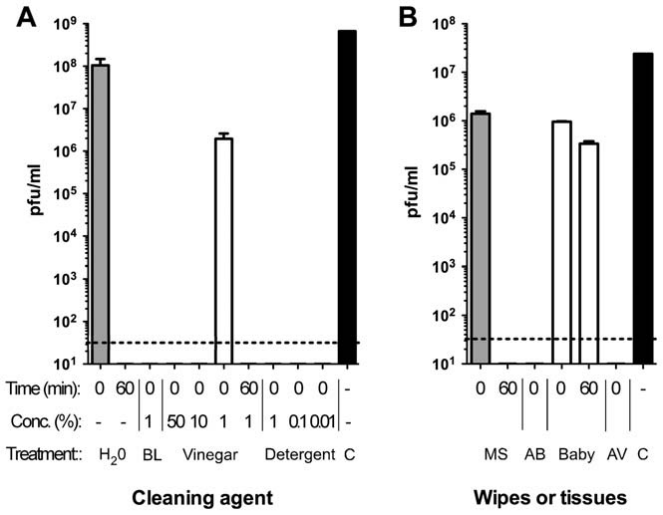
Effect on virus viability following treatment with the cleaning agents at various time points. A. Treatment with liquid household cleaning agents. BL, bleach; Detergent, washing up detergent; C, control (input virus). B. Treatment with wipes and tissues. MS, multisurface wipes; AB, anti-bacterial wipes; AV, anti-viral tissues. Data are derived from triplicate assays: error bars represent the standard error of the mean.

**Figure 2:**
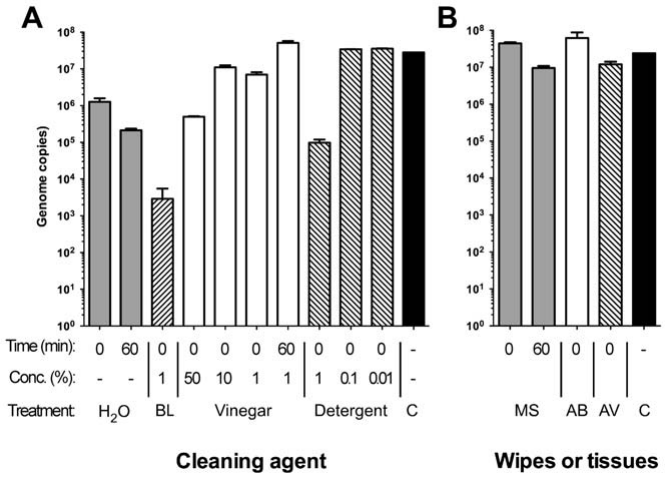
Effect on genome copy number following treatment with the cleaning agents at various time points. A. Treatment with liquid household cleaning agents. BL, bleach; Detergent, washing up detergent; C, control (input virus). B. Treatment with wipes and tissues. MS, multisurface wipes; AB, anti-bacterial wipes; AV, anti-viral tissues. Data are derived from triplicate assays: error bars represent the standard error of the mean.

A British Standard [1] was adapted to assess the ability of various household cleaning agents to kill or reduce the infectivity of an H1N1 human influenza virus A/PuertoRico/8/34 (PR8, H1N1, Cambridge lineage). We reasoned that routine domestic cleaning often uses hot water, and during an informal experiment gauged that many people ran ‘hand-hot’ water to a temperature of about 55°C; accordingly virus was mixed with water at this temperature, containing varying concentrations of bleach, washing up liquid and malt vinegar. Hot water alone was used as the baseline control. Samples were assayed either immediately (“0” minutes) as a measure of rapid inactivation or after 60 minutes to simulate prolonged contact and allow comparability with previous studies using this time cut-off. Plaque assays were used to assess the viability of the virus at set time points post incubation and quantitative RT-PCR was used to assay the effect of the cleaning agents on viral genome. The outcome of the plaque assays indicated that rapid treatment of the virus with hot water had little effect on the virus, reducing the titre by around two-fold, but prolonged incubation at 55°C abolished detectable infectivity. However, the addition of any of 1% bleach, 50% and 10% malt vinegar and 1%, 0.1% and 0.01% washing up liquid were all effective at rapidly reducing viable virus below the limit of detection, while a low concentration of vinegar (1%) was no more effective than hot water alone (Figure 1A). In contrast to the plaque assay results, most agents were ineffective at reducing the number of detectable genome copies as determined by RT-PCR, with only bleach having a significant effect (Figure 2A). The data for the plaque assays and RT-PCR assays are compiled in Tables S1 and S2. Thus, while a strong oxidizing agent such as bleach is effective at reducing both genome detection and virus infectivity, low pH and detergent are equally efficacious virucidal agents. These results also indicate that whilst vinegar and detergent disrupt the viral envelope proteins reducing infectivity, only bleach disrupts the viral genome.

### Wipes and Tissues

Surface cleaning wipes and antiviral tissues were assayed by a further adaptation of the BSEN14476:2005 Standard in which a single wipe to be tested was rinsed multiple times in room temperature sterile water. The resulting solution was filtered and used in the assay. Solubilised active ingredients from toddler wipes had a relatively small effect on virus viability, similar to treatment with hot water, causing around a 50-fold drop in titre even after 60 minute incubation (Figure 2A). Extracts from multisurface wipes cause a similar immediate reduction in virus infectivity, but on prolonged incubation showed a complete virucidal effect. Both the anti-bacterial wipe solution and the anti-viral tissue solution completely abrogated virus infection immediately after mixing with the virus (Figure 2A). However, viral genomes were detected in similar abundance by RT-PCR after all treatments with every wipe or tissue studied (Figure 2B, see also Table S2 for a comparison of numerical data). Thus, while all types of wipe tested had some antiviral effect, they varied considerably in the magnitude and rapidity of their virucidal effects.

## Discussion

The aim of this work was to identify commonly available cleaning agents and wipes which might be used during a pandemic situation to ensure household surfaces are free of viable influenza virus. By using hand-hot water (55°C) and concentrations of washing up liquid (0.1% to 0.01%) commonly used for domestic ‘washing up’ we attempted to create ‘real life’ conditions. However we acknowledge the tension in all such experiments between ‘real life’ assessments and the use of standard, reproducible internationally accepted assays. Although our study could not assess mechanical wiping effects, had we done so, it would have been difficult to assess our results in relation to ‘real life’ conditions, because wiping is carried out differently by individuals and the potential variability is considerable. Our work does not rule out a potentially important effect of physical wiping, though in real life this might also spread viruses further. A number of the agents tested were extremely efficient at killing the virus. These included 1% bleach, 10% malt vinegar, 0.01% washing up liquid, anti-bacterial wipes and anti-viral tissues. Some of these agents are relatively cheap and make for readily available, easy to use disinfectant products suitable for use in the home, even in low resource settings. The bleach used contains sodium hypochlorite and sodium hydroxide. The Food and Agricultural Organisation (FAO) of the United Nations recommends 2% sodium hydroxide (NaOH) for decontamination of animal housing equipment and machinery vehicles in order to be effective against avian influenza viruses [2]. Little work has been carried out to investigate the specific effects of NaOH against influenza viruses. However, there is evidence that treatment with 1% NaOH can reduce an A/H1N1 virus titre by up to 10^6^ EID_50_/0.2 ml [3]. Sodium hypochlorite is a chlorine-containing compound and the disinfectant nature of such agents arises due to the formation of hypochloric acid in water [4]. The WHO recommend 1% sodium hypochlorite (which contains 0.05% or 500 mg/L free chlorine) to disinfect surfaces and medical equipment [5]. Recent work also suggests that sodium hypochlorite at 750 ppm (750 mg/L) is capable of inactivating a low-pathogenicity avian influenza virus [6]. Further data suggests that other avian influenza viruses, such as A/H5N1, are inactivated by extremely low concentrations of chlorine (0.52–1.08 mg/L, [7]. Our data suggest that 1% household bleach, which equates to 0.05% sodium hypochlorite, are sufficient for the inactivation of human influenza viruses.

All dilutions of washing up liquid tested (down to 0.01%) inactivated the virus. Undiluted, this product contains 1–5% denatured ethanol, 15–30% ionic detergents and 5–15% non-ionic detergents. In a separate informal experiment we determined that a typical bowl of fresh ‘washing up’ water is likely to contain 0.1% to 0.01% washing up liquid. Although the alcohols have a denaturing effect on the viral proteins [4], at the concentrations used here it is most likely that the detergents are the active ingredients, acting to disrupt the viral envelope.

Vinegar is a commonly stocked household product, suitable for culinary use and also used for stain removal and other household cleaning. Malt vinegar (4–8% acetic acid) was effective down to a dilution of 10%. Previously 5% acetic acid has been demonstrated to be effective at inactivating an A/H7N2 strain of influenza [6] and it has been known for some years that acid-based media cause inactivation and aggregation of HA glycoprotein spikes and virus, by triggering the low pH-dependent conformational change in the HA that normally only occurs in late endosomes. [8].

Warm water is frequently used in the home to rinse surfaces and dishes. However, the data clearly show that, when used alone, it is ineffective in killing enveloped viruses, unless incubated with them for extended periods of time. Heating at 56°C of an A/H7N2 influenza strain for 30 minutes was shown to be effective at inactivating the virus [9]. However, there are conflicting data which demonstrate that A/H7N3 avian influenza viruses can withstand 56°C warm water incubation for up to 60 minutes [10]. All the liquid cleaning agents were diluted in warm water which may therefore have had a synergistic effect. However, due to the lack of fast killing with warm water alone, it is highly likely that it was the active ingredients in the cleaning agents which exerted a rapid virucidal effect.

The branded anti-bacterial wipes and anti-viral tissues were encouragingly effective at inactivating the virus. The branded anti-bacterial wipes contain butoxypropanol (1–5%) and ethanol (5–10%). The branded anti-viral tissues contain citric acid (7.81%) as the active ingredient. *In vitro* tests demonstrated that citric acid based buffer solution nasal sprays reduced the titre of an influenza A Sydney/5/97 (H3N2) influenza strain by up to 3 logs after 1 minute contact time [11]. Citric acid works in a similar manner to acetic acid, inducing the low pH transition in the viral HA protein thus rendering it unable to mediate cell entry.

The toddler wipes and multi surface wipes which were markedly less effective contain <5% surfactants, compounds recommended for use against influenza because they disrupt the integrity of the lipid virus envelope [12]. Our data indicate that the surfactants in the wipes are not present in high enough concentration to inactivate PR8 in under 60 minutes. The toddler wipes contain citric acid in common with the highly effective antiviral tissues; however the concentration in the former is not specified and may well be too low to have a substantial effect on virus infectivity.

Most of the cleaning agents had little effect on genome copy number. However, 1% bleach reduced copies of the genome by over a thousand fold. In another study, treatment of avian influenza viruses with 1% sodium hypochlorite resulted in no detectable RNA [13]. A high concentration of washing up liquid (1%, which contains alcohol and surfactants) showed a 3 log drop in genome copies compared to 1 log with 0.1 or 0.01% washing up liquid. Alcohol based hand gels have been demonstrated to reduce A/H1N1 down to only 100 virus copies/µl [14].

### Conclusions

The virus envelope not only protects the genome and core virion proteins but also acts as a vector to transfer genome between host cells. Disruption of the envelope either by lipid attack (causing disintegration) or protein denaturation (preventing fusion to host cells) inhibits the virus being transmitted to a new host. Active ingredients in a number of the cleaning agents, wipes and tissues tested were able to target the influenza envelope and render the virions non viable. Some of these agents were also capable of destroying the viral genome, in particular bleach. In the context of the on-going pandemic and the control of interpandemic influenza in the home, it is possible to conclude that in a household setting, simple, readily available products such as 1% bleach, 10% vinegar and 0.01% washing up liquid all make convenient, easy to handle killing agents for influenza virus A/H1N1. These findings can be readily translated into simple public health advice, even in low resource settings. The public do not need to source more sophisticated cleaning products than these; notwithstanding, wipes with a claimed antiviral or antibacterial effect are also likely to be highly effective. However, caution should be exercised with non-microbicidal ‘cleansing’ wipes and toddler wipes containing only low concentrations (<5%) of surfactants as these appear to have less anti-influenza action. It may be appropriate for families intending to use wipes to reduce influenza transmission in the home, to be advised not to assume that all have equal anti-influenza properties and to be encouraged to select brands with certified or validated anti-viral or antibacterial properties.

## Materials and Methods

### Cleaning Agents Tested

The following agents were tested: 1% bleach (Domestos®: Unilever, UK); 50%, 10% & 1% malt vinegar (Sainsburys, UK); 1%, 0.1% and 0.01% washing up liquid (Original Fairy Liquid®, Proctor and Gamble, UK). Water at 55°C was included as a control. The agents were also diluted in this, since it represented a comfortable hot water temperature which people might use in a domestic household.

### Wipes and Tissues Tested

Branded antibacterial wipes (Flash Strongweave®: Proctor and Gamble, UK); Branded multi-surface furniture wipes (Mr Sheen®: Reckitt and Benckiser, UK); Branded toddler wipes (Toddler wipes: Sainsburys, UK); Branded anti-viral tissues (Kleenex®: Kimberly-Clark, USA).

### Viruses and Cells

Human influenza virus A/PuertoRico/8/34 (H1N1, Cambridge lineage) was grown in embryonated eggs and titrated in MDCK cells as previously described [15], [16]. Stocks at a titre of 1.3–1.9×10^8^ pfu/ml were used. This and subsequent dilutions in distilled H_2_0 were within the range of concentrations found in nasal secretions [17]. Madin Darby Canine Kidney cells (MDCKs) obtained from the European Collection of Cell Cultures were used in the virus plaque assays. They were maintained in Dulbeccos Modified Eagles Medium (DMEM, Gibco, UK) containing 2 mM glutamine, 10% foetal calf serum, 100 µg/ml penicillin and 100 µg/ml streptomycin (Invitrogen). Cells were split using 0.25% trypsin/EDTA and seeded at 1.6×10^6^/well in six-well tissue culture plates for plaque assays.

### Nucleic Acid Extraction and qRT-PCR

0.5 ml samples were processed using the NucliSENS® easyMAG system (Biomerieux), with an elution volume of 60 µl. As an internal control, bacteriophage MS2 (ATCC 15597-B1) at approximately 4000 pfu was included in each sample [18].

### qRT-PCR

All primers and probes were obtained from Metabion, with the exception of the MGB probe which was obtained from Applied Biosystems (ABI). The enzyme and buffers used were from the Invitrogen Superscript™ III Platinum kit. Primers and probes used in the RT-PCT were as follows:

MS2-F and MS2-R: Forward and reverse primers binding to target sequences on the internal MS2 bacteriophage control, 20 pmol/µl each.

MS2F: 5′ TGG CAC TAC CCC CTC TCC GTA TTC ACG-3′.

MS2R: 5′ GTA CGG GCG ACC CCA CGA TGAC-3′.

MS2 Rox/Probe: Taqman® probe that hybridizes to a target sequence on the MS2 internal control, 10 pmol/µl. 5′ Rox- CAC ATC GAT AGA TCA AGG TGC CTA CAA GC–BHQ2 3′

AM-F and AM-R: Generic forward and reverse primers respectively binding to target sequences in the Influenza A matrix gene, enabling amplification of the matrix RNA as a marker of virus genome copies 20 pmol/µl each.

AMF: 5′ GAG TCT TCT AAC MGA GGT CGA AAC GTA-3′.

AMR: 5′ GGG CAC GGT GAG CGT RAA-3′.

AM Probe: Taqman® probe that hybridizes to a specific target sequence on the Influenza A matrix gene, 10 pmol/µl. 5′ VIC-TCC TGT CAC CTC TGA C-MGBNFQ-3′.

Each 20 µl RT-PCR reaction contained 4.4 µl water, 12.5 µl 2 x buffer, 0.1 µl each MS2 primers, 0.2 µl MS2 probe, 0.5 µl AMF, 1 µl AMR, 0.4 µl AM probe, and 0.8 µl Superscript™ III Platinum one-step enzyme.

DNA Matrix plasmid standards of known concentration from −5 (4.8×10^6^) to −10 (4.8×10^1^) were used to construct a standard curve, from which the genome copies in the samples could be calculated.

RT-PCR was performed on a Rotor-Gene™ 6000 (Corbett Research) real-time DNA detection system as follows: Incubation at 50°C for 30 minutes, followed by a further incubation at 95°C for 2 minutes and then 45 cycles of amplification with denaturation at 95°C for 15 minutes and annealing and extension at 60°C for 1 minute. Acquisition was on the Joe and Rox channels at each cycle.

### Virus Killing Assay: Cleaning Agents

This assay was based upon British Standard BS EN 14476:2005. 60 µl of PR8 virus stock was diluted (1∶10) into 150 µl 10% BSA solution and 1290 µl Serum Free Media (DMEM + glutamine + penicillin/streptomycin). To simulate the household situation, autoclaved (15 minutes at 121°C) tap water was used as the diluent. When using the tap water alone as a reagent, 15 ml was added to a 50 ml tube and placed in a water bath set to 55°C. For all other cleaning agents, dilutions were made in autoclaved tap water. For all reagents tested, a separate 15 ml tube containing autoclaved tap water was heated to 55°C in the water bath. This was used as the control experiment. The 15 ml of cleaning agent was added to the diluted PR8 (see above) to begin the assay. Samples of the PR8/disinfectant test solution were taken, in triplicate, at 0 (i.e. immediately), & 60 mins. The test solution was removed in order to sample the virus but replaced into the water bath for incubation until later sampling time points. For each sample, 450 µl was taken, snap frozen on dry ice and stored at −70°C. These aliquots were used in the plaque assays to assess the effect of each agent on virus viability. At the same time points, 500 µl was added to 2 ml lysis buffer for RT-PCR and stored at −20°C. This was also done in triplicate. For each cleaning agent experiment, including water alone, a positive control experiment was performed to ensure that the plaque assay method worked under these conditions. A 1∶25 dilution of PR8 stock was made by adding 10 µl PR8 into 25 µl 10% BSA solution and 215 µl Serum Free Media (SFM, DMEM + glutamine + penicillin/streptomycin) in a 15 ml tube. 2 ml of 55°C autoclaved tap water was added to the PR8 control dilution to begin the assay. Immediately, 450 µl was sampled, snap frozen and stored at −70°C for plaque assay. At the same time, 500 µl was added to NucliSENS lysis buffer for RT-PCR.

### Virus Killing Assay: Wipes/Tissues

The PR8 stock was diluted as for the liquid cleaning agent experiments. In a Biological Safety Cabinet, a wipe/tissue was removed from its packaging and rinsed 3 times in 75 ml of cold sterile water. 15 ml of the wipe/tissue solution was taken up into a 20 ml syringe and filtered through a Ministart® filter (0.45 µm) to remove wipe particles in the solution. The procedure was repeated for the three wipes and tissue product.

15 ml of wipe/tissue solution was added to the diluted PR8 (see above) to begin the assay. Samples of this solution were taken, in triplicate, at 0 (immediately) and 60 mins. For each sample, 450 µl was taken, snap frozen using dry ice and stored at −70°C. At the same time points, 500 µl was added to 2 ml lysis buffer for RT-PCR.

As with the liquid cleaning agents, positive controls were included. Unlike the controls for the liquid agents where PR8 was treated with autoclaved 55°C tap water, the controls for the wipe experiments were treated with sterile water.

### Plaque Assay

Plaque assays were carried out in 6 well culture dishes containing confluent MDCK cells overlaid with 2 ml of a mixture of 50% serum free medium, 50% Avicel (Signet Chemical Corporation, India), 1 µg/ml Worthington's Trypsin and 0.14% BSA, where the volume of each component was adjusted based on the number of plaques assays performed [16], [19]. The cells were then incubated at 37°C for 48 hours without agitation, before fixation with formal saline and staining using 1% Toluidine Blue.

## Supporting Information

Table S1 Assessment by plaque assay of the effect of liquid household cleaning agents and wipes on influenza virus A viability.
(0.04 MB DOC)

Table S2 Assessment by RT-PCR of the effect of liquid cleaning agents, wipes, and tissues on influenza virus A genome copy number.
(0.04 MB DOC)
