# Supplementary material for: Effectiveness of Common Household Cleaning Agents in Reducing the Viability of Human Influenza A/H1N1
Source: PLoS One. 2010 Feb 1;5(2):e8987. doi: 10.1371/journal.pone.0008987 (PMC2813869; doi:10.1371/journal.pone.0008987)
Supplement: Table S1 — Assessment by plaque assay of the effect of liquid household cleaning agents and wipes on influenza virus A viability. (0.04 MB DOC) [file pone.0008987.s001.doc]

**Table S1. Assessment by plaque assay of the effect of liquid household cleaning agents and wipes on influenza virus A viability**.

| **Cleaning agent** | **Time (mins)** | **Virus titre (pfu/ml)** | **Average titre**  **(pfu/ml)** |
| --- | --- | --- | --- |
| **55 °C water** | 0    60 | 7.5 x 10^7^ 5.0 x 10^7^ 1.9 x 10^8^  0 0 0 | 1.1 x 10^8^  0 |
| **1 % bleach** | 0 | 0 0 0 | 0 |
| **50 % malt vinegar** | 0 | 0 0 0 | 0 |
| **10 % malt vinegar** | 0 | 0 0 0 | 0 |
| **1 % malt vinegar** | 0    60 | 1.3 x 10^6^ 2.6 x 10^6^  NT  0 0 0 | 2.0 x 10^6^  0 |
| **1 % washing up liquid** | 0 | 0 0 0 | 0 |
| **0.1 % washing up liquid** | 0 | 0 0 0 | 0 |
| **0.01 % washing up liquid** | 0 | 0 0 0 | 0 |
| **Multi-surface wipes** | 0  60 | 1.1 x 10^6^ 1.4 x 10^6^ 1.7 x 10^6^  0 0 0 | 1.4 x 10^6^  0 |
| **Toddler wipes** | 0  60 | 9.4 x 10^5^ 9.4 x 10^5^ 1 x 10^6^  2.5 x 10^5^ 3.8 x10^5^ 3.8 x 10^5^ | 9.6 x 10^5^  3.4 x 10^5^ |
| **Anti-bacterial wipes** | 0 | 0 0 0 | 0 |
| **Anti-viral tissues** | 0 | 0 0 0 | 0 |
