# Supplementary material for: Effectiveness of Common Household Cleaning Agents in Reducing the Viability of Human Influenza A/H1N1
Source: PLoS One. 2010 Feb 1;5(2):e8987. doi: 10.1371/journal.pone.0008987 (PMC2813869; doi:10.1371/journal.pone.0008987)
Supplement: Table S2 — Assessment by RT-PCR of the effect of liquid cleaning agents, wipes, and tissues on influenza virus A genome copy number. (0.04 MB DOC) [file pone.0008987.s002.doc]

**Table S2: Assessment by RT-PCR of the effect of liquid cleaning agents, wipes and tissues on influenza virus A genome copy number.**

| **Cleaning agent** | **Time (mins)** | | **Genome copy number** | **Average genome copy number** |
| --- | --- | --- | --- | --- |
| **55 °C water** | 0    60 | 7.3 x 10^5^ 1.8 x 10^6^ 1.3 x 10^6^  2.4 x 10^5^ 2.3 x 10^5^ 1.7 x 10^5^ | | 1.3 x 10^6^  2.1 x 10^5^ |
| **1 % bleach** | 0 | 3.3 x 10^2^ 8.1 x 10^3^ 2.7 x 10^2^ | | 2.9 x 10^3^ |
| **50 % malt vinegar** | 0 | 5.1 x 10^5^ 4.6 x 10^5^ 5.2 x 10^5^ | | 5.0 x 10^5^ |
| **10 % malt vinegar** | 0 | 8.2 x 10^6^ 1.2 x 10^7^ 1.3 x10^7^ | | 1.1 x 10^7^ |
| **1 % malt vinegar** | 0    60 | 4.9 x 10^6^ 7.5 x 10^6^ 8.6 x 10^6^  6.2 x 10^7^ 5.1 x 10^7^ 4.1 x 10^7^ | | 7 x 10^6^  5.1 x 10^7^ |
| **1 % washing up liquid** | 0 | 1.4 x 10^5^ 7.4 x 10^4^ 7.9 x 10^4^ | | 9.8 x 10^4^ |
| **0.1 % washing up liquid** | 0 | 3.4 x 10^7^ 3.4 x 10^7^ 3.5 x 10^7^ | | 3.4 x 10^7^ |
| **0.01 % washing up liquid** | 0 | 3.5 x 10^7^ 3.6 x 10^7^ 3.6 x 10^7^ | | 3.6 x 10^7^ |
| **Multi-surface wipes** | 0  60 | 3.9 x 10^7^ 4.9 x 10^7^ 4.5 x 10^7^  1.2 x 10^7^ 7.5 x 10^6^ 9.0 x 10^6^ | | 4.4 x 10^7^  9.5 x 10^6^ |
| **Toddler wipes** | 0  60 | 6.8 x 10^5^ 7.6 x 10^5^ 8 x 10^6^  6.6 x 10^5^ 5.8 x10^5^ 8.9 x 10^5^ | | 7.5 x 10^5^  7.1 x 10^5^ |
| **Anti-bacterial wipes** | 0 | 9.0 x 10^7^ 7.5 x 10^7^ 8.5 x 10^7^ | | 8.3 x 10^7^ |
| **Anti-viral tissues** | 0 | 8.9 x 10^6^ 1.1 x 10^7^ 1.6 x 10^7^ | | 1.2 x 10^7^ |
